# TLK1B promotes repair of DSBs via its interaction with Rad9 and Asf1 Caroline Canfield, Justin Rains, and Arrigo De Benedetti

**DOI:** 10.1186/1471-2199-10-110

**Published:** 2009-12-20

**Authors:** Caroline Canfield, Justin Rains, Arrigo De Benedetti

**Affiliations:** 1 Department of Biochemistry and Molecular Biology and the Feist-Weiller Cancer Center, Louisiana State University Health Sciences Center, Shreveport, LA 71130, USA

## Abstract

**Background:**

The *Tousled-like kinases *are involved in chromatin assembly, DNA repair, transcription, and chromosome segregation. Previous evidence indicated that TLK1B can promote repair of plasmids with cohesive ends in vitro, but it was inferred that the mechanism was indirect and via chromatin assembly, mediated by its interaction with the chromatin assembly factor Asf1. We recently identified Rad9 as a substrate of TLK1B, and we presented evidence that the TLK1B-Rad9 interaction plays some role in DSB repair. Hence the relative contribution of Asf1 and Rad9 to the protective effect of TLK1B in DSBs repair is not known. Using an adeno-HO-mediated cleavage system in MM3MG cells, we previously showed that overexpression of either TLK1B or a kinase-dead protein (KD) promoted repair and the assembly of Rad9 in proximity of the DSB at early time points post-infection. This established that it is a chaperone activity of TLK1B and not directly the kinase activity that promotes recruitment of 9-1-1 to the DSB. However, the phosphorylation of Rad9(S328) by TLK1B appeared important for mediating a cell cycle checkpoint, and thus, this phosphorylation of Rad9 may have other effects on 9-1-1 functionality.

**Results:**

Here we present direct evidence that TLK1B can promote repair of linearized plasmids with incompatible ends that require processing prior to ligation. Immunodepletion of Rad9 indicated that Rad9 was important for processing the ends preceding ligation, suggesting that the interaction of TLK1B with Rad9 is a key mediator for this type of repair. Ligation of incompatible ends also required DNA-PK, as addition of wortmannin or immunodepletion of Ku70 abrogated ligation. Depletion of Ku70 prevented the ligation of the plasmid but did not affect stimulation of the fill-in of the ends by added TLK1B, which was attributed to Rad9. From experiments with the HO-cleavage system, we now show that Rad17, a subunit of the "clamp loader", associates normally with the DSB in KD-overexpressing cells. However, the subsequent release of Rad17 and Rad9 upon repair of the DSB was significantly slower in these cells compared to controls or cells expressing wt-TLK1B.

**Conclusions:**

TLKs play important roles in DNA repair, not only by modulation of chromatin assembly via Asf1, but also by a more direct function in processing the ends of a DSB via interaction with Rad9. Inhibition of Rad9 phosphorylation in KD-overexpressing cells may have consequences in signaling completion of the repair and cell cycle re-entry, and could explain a loss of viability from DSBs in these cells.

## Background

The gene *Tousled *of *Arabidopsis thaliana *encodes a protein kinase which, when mutated, results in abnormal flower development [1]. This was proposed to be linked to a replicative defect during organogenesis, but which may also result from failure to protect the genome from UV damage [2,3], resulting in mitotic aberrations [4-6]. Two *Tousled *genes (TLK1 and TLK2) were identified in mammals [7,8], and were confirmed as encoding kinases. Few physiologic substrates of *Tousled *like kinases (TLKs) have been identified, namely Asf1 [9], histone H3 [10], Aurora B [5], and more recently Rad9 in mammalian cells [11] and two mitotic kinesins in Trypanosomes [12]. This suggested a function in chromatin assembly [13] during transcription [14,2], DNA repair [3,15], and condensation of chromosomes at mitosis [4]. Evidence also exists about a link between TLKs and a DNA damage relay [16]. This can be inferred from research that shows that the activity of TLK1 is inhibited by IR and genotoxins [16]. The inhibition is mediated by ATM via Chk1 by direct phosphorylation at S695 [17]. These findings identify a functional cooperation between ATM and Chk1 in propagation of a checkpoint response mediated by transient inhibition of TLK1, which may regulate processes involved in chromatin assembly [16]. A splice variant of TLK1 that is translated upon genotoxic stress [18], TLK1B, has invoked interest because of its established role in cell survival from DNA damage [3,10,15]. TLK1 and TLK1B share identity in the catalytic domain and hence interact with mostly the same substrates, and we often refer to them as TLK1/1B in this respect. Earlier studies showed that elevated expression of TLK1B promotes cell survival after radiation or doxorubicin by facilitating DNA repair [15]. These initial studies suggested that the role of TLK1B in radioprotection could be mediated through Asf1, leading to changes in chromatin disassembly/assembly coupled to repair [3,15]. In fact, initial studies have explored the role of Asf1 and TLK1/1B by in vitro chromatin assembly on a plasmid [19,15]; and a possible role of TLKs in repair was reviewed in [20]. More recently however, the identification of Rad9 as a substrate of TLK1B suggests that TLK1B can affect DSB processing through modulation of 9-1-1 activity. Rad9, Rad1, and Hus1 form a trimeric complex (termed 9-1-1) that is structurally similar to the Proliferating Cell Nuclear Antigen [21] "sliding-clamp", which encircles the DNA conferring processivity to polymerases [22-24]. 9-1-1 assembles in a complex at sites of damage [25], and it is the genotoxin-activated RFC-Rad17 "clamp loader" that locks the 9-1-1 complex onto DNA [25]. The 9-1-1 then serves as a scaffold for assembly of DNA repair proteins, Flap endonuclease [26,27], DNA polymerase β [28], DNA ligase 1 [29], and DNA glycosylase MutY [30], in addition to aiding processing of the DNA ends by its own exonucleolytic activity [31-33]. Accordingly, Rad9 is held to be involved in translesion repair synthesis [34], mismatch repair [35], removal of 8-oxoguanine [36], and NHEJ and HRR [37,38]. We showed that TLK1B phosphorylates Rad9 at S328, and that this phosphorylation appears to play a role in resumption of cell cycle after IR. However, TLK1B had also a function as a chaperone for Rad9 assembly at DSBs that was independent of its kinase function [11]. We now address in more details, in vitro and in intact cells, the role of TLK1B in DSB repair, in particular in relation to Rad9 functionality.

## Results and Discussion

### Repair of DSB in vitro

We previously described a system with nuclear extract of MM3MG cells that allows to monitor repair of a plasmid that is cut with EcoRI, which leaves a cohesive 5' overhang [15]. Repair of plasmid was seen on EtBr-stained gels as formation of high mobility forms, which are the result of religation and simultaneous assembly of nucleosomes on the template. The increased mobility of the plasmid compared to the linear form is due to a decrease in the linking number by formation of chromatin, resulting in supercoiling - only covalently closed plasmid can be supercoiled. We have more recently analyzed the predominant repaired junctions obtained from this in vitro repair system, by transformation of bacteria and sequencing of the plasmids rescued from several clones. In most cases examined, the repair had been a high-fidelity ligation which resulted in reconstitution of the EcoRI site by simple annealing and joining of the ends (16/20 clones). Two different clones displayed a deletion of the 4-base overhang, and the two remaining had a 3-base fill-in and deletion of the last base. We had shown that the addition of TLK1B hastened the repair and supercoiling of the plasmid [15], which at the time was attributed to the known interaction of TLK1/1B with Asf1. However, we had not examined this directly. In particular, it was not known if depletion of Asf1 would result in loss of ligation and supercoiling. This seemed possible since Asf1, by virtue of its activity on nucleosome assembly, could perhaps promote repair indirectly by compacting the plasmid and bringing the ends in juxtaposition. Thus, we needed to test the effect of Asf1 depletion on ligation of cohesive ends.

#### Depletion of Asf1

**Figure 1:**
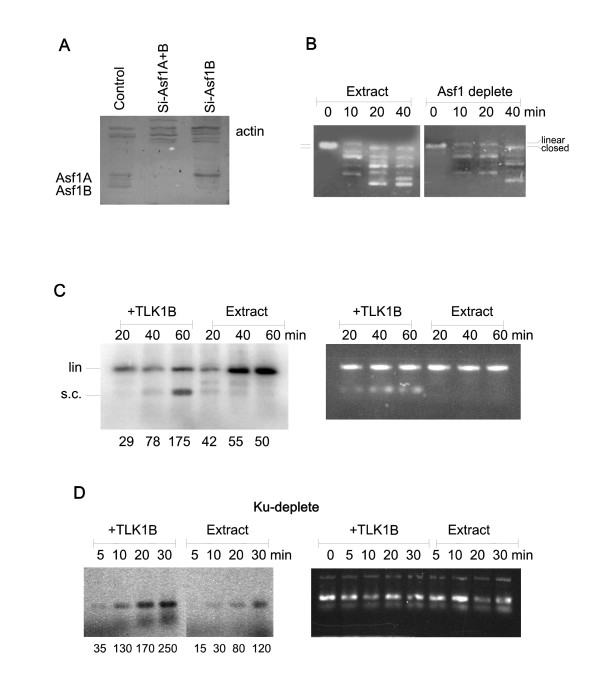
**Repair of linearized plasmids in cell extract**. A) *Preparation of Asf1-depleted cell extract*. Depletion of Asf1A and Asf1B with respective siRNAs was monitored by western blot. B) *Ligation of cohesive (EcoRI ends) and role of Asf1*. The reaction was assembled as described in [15,3]. Plasmid cut with EcoRI was incubated with Asf1-depleted extract or control, to monitor end-joining repair coupled with nucleosome assembly. The positions of the linearized and closed/relaxed forms of the plasmid are marked. C) *Ligation of EcoRI/EcoRV-cut plasmid*. The reactions contained 5 μCi [α-^32^P]dATP (0.1 mM final concentration) to label the plasmid by endogenous polymerases. In the left and right panels (autorad and EtBr), the stimulation of plasmid religation/supercoiling by added TLK1B is shown. The position of the linearized and ligated/supercoiled form is shown. Quantitation of the autorad in pixels (supercoiled form) is shown below each lane. D) *Labeling by fill-in in Ku-depleted extract*. The effect of TLK1B on end-labeling is shown in the absence of ligation. Quantitation of the autorad in pixels is shown below each lane.

We previously described the immunodepletion of Asf1 with an antibody from CIM (Antibody Core at Arizona State University [3]) but this antiserum is no longer available, and an antiserum from Santa Cruz contained a nuclease. Thus, we depleted Asf1 with siRNA. Successful depletion of Asf1B and Asf1A+B is shown in Fig. 1A, and we prepared nuclear extracts from these cells. Incubation of EcoRI-cut plasmid with nuclear extract resulted in ligation/supercoiling, which was seen as an array of faster-migrating, discrete bands (Fig. 1B). Depletion of Asf1 had some effect on supercoiling (compare the fastest migrating band at 20 and 40 min), but it had only modest effect on religation of the ends. Quantitative analysis showed that conversion of the linear form to circular/relaxed and then supercoiled was complete after 20 min in control extract, but not until 40 min in Asf1-depleted. Hence, Asf1, albeit likely involved, is not essential for these repair reactions nor for supercoiling, consistent with a similar result we had seen for repair of UV-damaged plasmids [3]. Likely, another histone chaperone can substitute for Asf1 in nucleosomes formation in vitro, although less rapidly. In previous work we had found that Asf1 is primarily involved in the in vitro assembly of nucleosomes [3,15] and consistent with this view, in yeast, Asf1 appears to be involved in subsequent steps to DSB repair and in reformation of chromatin at the repaired junctions [39].

#### Ligation of incompatible ends

To test if the repair of incompatible ends was also stimulated by TLK1B, we set out to probe if the ligation of a blunt end generated by EcoRV and one generated by EcoRI (both in the MCS of Bluescript) could be repaired in our system. The predominant form of repair of incompatible ends in mammalian extracts is blunt-end ligation, which is achieved by either fill-in or resection of the ends [40]. In rarer cases, cohesive-ends ligation occurs via more complex processing that results in short stretches of micro-homology [40]. We first determined if this nuclear extract system could repair the plasmid by fill-in of the EcoRI end, followed by blunt ligation to the EcoRV end. Fill-in of the EcoRI end by repair polymerases was monitored via incorporation of [α^32^P]dATP. In this case, the generation of a flush end at the EcoRI side could either take place by resection of the remaining 5'-ApA overhang or, less likely, by fill-in with residual dTTP that may be present in the nuclear extract. Successful end-joining and simultaneous plasmid supercoiling via formation of nucleosomes was monitored as an increased mobility (Fig. 1C). Since the supercoiled form is labeled, there must have been primarily a blunt-end joining of the filled site, as complete resection of the EcoRI end would have resulted in loss labeling. At least, this assay system is geared toward studying primarily such repair reaction. The addition of recombinant TLK1B resulted in significantly greater amounts of religated/supercoiled molecules than extract alone, as shown by examining the autoradiogram or the EtBr-stained gel. Transformation of bacteria yielded no colonies with linearized plasmid, but dozens with the extract-mediated repair/ligation. Analysis of the repaired junctions in plasmids from 10 clones revealed filling with two A's and the excision of the two remaining 5'A's. Also, under these repair conditions, there was one predominant religated, fast-migrating form of the plasmid. This is in contrast to what we normally see for repair of plasmids singly cut with EcoRI, which typically results in a ladder of topoisomeric forms - the result of the assembly of a dense array of nucleosomes (see panel B). A possible explanation for this different result is that simple ligation of cohesive ends is rapid compared to ligation of incompatible ends, thus allowing more time for the assembly of a dense array of nucleosomes, which are later resolved by endogenous toposiomerases and resulting in supercoiling.

#### The fill-in reaction is independent of ligation but may be a prerequisite for it

**Figure 2:**
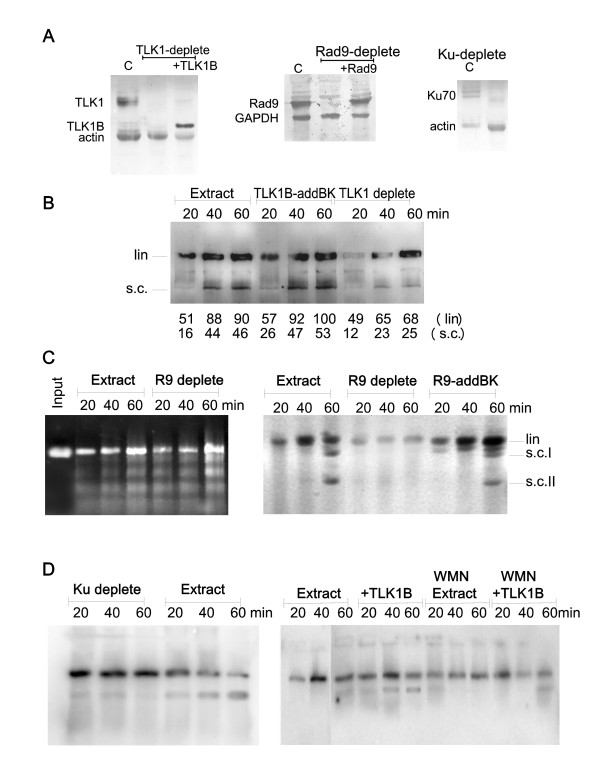
**Repair of linearized palsmids and dependence on TLK1, Rad9, and Ku70**. A) *Western blots of extracts depleted of TLK1, Rad9, and Ku70*. B) *Immunodepletion of TLK1 and add-back*. The effect of immunodepleting the endogenous TLK1 in labeling of the ends and ligation/supercoiling is shown. In the middle lanes, recombinant TLK1B was added back to the immunodepleted extract in an amount comparable to the endogenous TLK1 (see panel A). Note that this gel was exposed for longer than that in Fig. 1C to reveal the repaired forms more clearly. Quantitation of the autorad in pixels is shown below each lane. C) * Plasmid repair dependence on Rad9*. Where indicated, the extract was immunodepleted of Rad9. In the left panel, we monitored repair of plasmid linearized with EcoRI alone (cohesive ends repair). In the middle panel, we monitored labeling and religation/supercoiling of plasmid cut with EcoRI/EcoRV, and its dependence on Rad9. In the right panel, Rad9 was added back. The position of linear and two supercoiled forms of the plasmids are indicated. D)* Plasmid repair dependence on Ku70 and DNA-PK*. The plasmid was in this case pre-labeled with Kleonw polymerase and [α-^32^P]dATP. Where indicated, the extract was immunodepleted of Ku (left panel), or the reaction was carried out in presence of 1 μM wortmainnin (right panel). Where indicated, we added TLK1B.

So far, it was unclear if the effect of TLK1B in repair was due to a more rapid fill-in (via Rad9) that may be a prerequisite for blunt-ends ligation or whether the effect was instead mediated by Asf1 by plasmid compaction (or due to both). To test this, the fill-in reaction and repair was studied in extract depleted of Ku70 (Fig. 1D and see also Fig. 2), as DNA-PK/Ku is required for ligation of such ends (reviewed in [41]. Hence, the fill-in reaction could be studied separately from the ligation/supercoiling effect. Under these condition, TLK1B stimulated the labeling of the ends (fill-in), whereas almost no ligation, inferred by supercoiling, could be seen (Fig. 1D).

#### Role of TLK1 and TLK1B

Whereas the addition of TLK1B, the form that is induced in presence of DSBs [18], stimulated repair/supercoiling, it was not known if TLK1, the constitutively expressed form, is also important for DNA repair. To test this, we immunodepleted the extract of TLK1, and we monitored ligation/supercoiling on a plasmid cut with EcoRI and EcoRV. In Fig. 2A, we show western blots of extracts immunodepleted of TLK1, Rad9, and Ku70, which were then used in this study. In Fig. 2B, we show that depletion of TLK1 resulted in a small effect on plasmid ligation/supercoiling, although it appeared that the labeling of the ends also occurred more slowly (last 3 lanes). In fact, it seems that the reduction in supercoiling is proportional to the reduction in labeling, and likely through an effect of TLK1 on Rad9. Hence, in this case, the fill-in reaction may be stimulated by presence of TLK1, although not absolutely required. Adding back TLK1B restored the efficient labeling of the ends and increased ligation/supercoiling (Fig. 2B, middle lanes). We have failed to produce full-length recombinant TLK1, and hence we have not been able to study the effect of this larger isoform in add-back assays.

#### Role of Rad9 in repair in vitro

We propose that the effect of TLK1B in promoting plasmid repair is mediated by its specific interaction with Asf1 [3] and Rad9 [11]. But the evidence so far is that Rad9 is more directly affecting repair by aiding processing of the ends, seemingly by fill-in via recruitment of repair polymerases [31-33]. Thus, we first tested if the simple repair of cohesive ends was dependent on Rad9. Extract was immunodepleted of Rad9, and EcoRI-cut plasmid was added. Ligation and formation of supercoiled forms was assessed by gel electrophoresis and staining with EtBr. Clearly, the plasmid was rapidly religated in these conditions and assembled in nucleosomes; and presence of Rad9 was not needed for this type of repair/supercoiling (Fig. 2C, left panel). This should not be surprising, since the joining of this type of cohesive ends depends mostly on ligase 4/XRCC4 and the likely association of DNA-PK to the break [42,41], and not so much on the 9-1-1 complex. We then tested if the repair of incompatible ends does rely on Rad9. The repair reaction was carried on plasmid cut with EcoRI and EcoRV in presence of [α^32^P]dATP. Labeling of the plasmid and modest ligation and supercoiling was obtained with whole extract, but depletion of Rad9 inhibited both (Fig. 2C, right panel). Adding back Rad9 restored robust labeling of the ends and also more religated/supercoiled plasmid. Overall, this indicates that some repair function of Rad9, likely its aiding of fill-in repair [28], its own exonucleolytic activity [33], or its interaction with FEN1 [26] are important to process these types of ends to promote ligation.

#### Role of DNA-PK in ligation

Finally, we set out to test if the repair of incompatible ends depends on Ku70 and DNA-PKcs. This was a requirement for the experiment shown in Fig. 1D, and is also a control for the entire work, as end-joining normally requires DNA-PK [41]. In this case, to avoid possible differences in end-labeling efficiency in the different reactions, after cutting with EcoRI and EcoRV, Klenow polymerase was added with [α^32^P]dATP to pre-label the plasmid. The labeled plasmid was then resin-purified and added to extract immunodepleted of Ku70 or not. Depletion of Ku70 resulted in loss of ligation/supercoiling (Fig. 2D, left panel), indicating that formation of a synaptic complex consisting of the plasmid ends associated with DNA-PK [43] is a pre-requisite for religation in this system. To further study if DNA-PKcs was important in such conditions, wortmannin (WMN) was included in these reactions. WMN inhibits PI3K members, including DNA-PKcs, ATM and ATR. However, research with more specific inhibitors of DNA-PKcs and the use of extracts from cells deficient in DNA-PKcs have strongly suggested that DNA-PK is the primary enzyme involved in the in vitro repair of these types of ends [42]. The addition of WMN almost completely prevented the formation of religated/supercoiled forms, even when TLK1B was added to the extract (Fig. 2D, right panel). These experiments strongly indicate that the processing of ends prior to ligation requires Rad9 and DNA-PK.

#### Blunt ends is a prelude to ligation of incompatible ends

**Figure 3:**
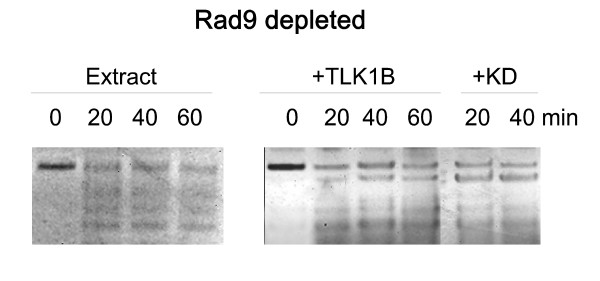
**Ligation and supercoiling does not depend on Rad9 when the ends are already blunt**. The plasmid cut with EcoRI and EcoRV was pre-labeled and filled-in with [α-^32^P]dATP and dTTP. Rad9-depeleted extract was used for these repair reactions, and the effect of the addition of TLK1B or the KD was also shown.

To establish if the main effect of Rad9 was to polish the ends to create conditions suitable for blunt-end ligation, the plasmid was filled-in with Klenow polymerase and [α^32^P]dATP and dTTP, and incubated with extract depleted of Rad9. As seen in Fig. 3, the extract was in this case capable of promoting ligation/supercoiling, confirming that the generation of flush ends is really the limiting step for repair of incompatible ends in these reactions. The addition of TLK1B or the kinase-inactive mutant (KD) stimulated the formation of the more highly supercoiled forms. This effect can clearly be attributed to the effect of TLK1B on Asf1, and is consistent with our previous report that it is the chaperone function of TLK1B, not its kinase activity, that is responsible for stimulation of nucleosome formation [11]. Furthermore, TLK1B can counteract Asf1's inhibition of H3/H4 tetramerization in the absence of ATP [44]

### Repair of a single DSB in intact cells

**Figure 4:**
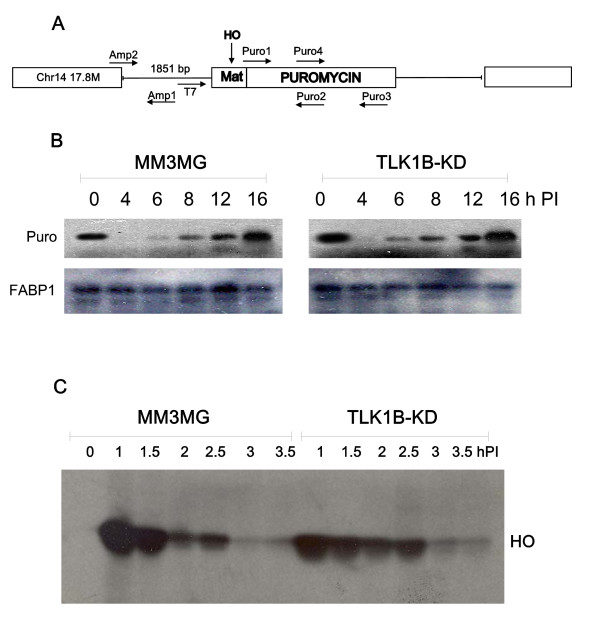
**Genomic HO-mediated cleavage and repair**. A) *Diagram of the Mat cassette integrated at chromosome 14*. These cell lines were described in [11]. B) *Time course Post Infection (PI) with adeno-HO*. Presence of the DSB was followed by PCR, probing for a ~500 bp product generated with primers T7 and Puro2 flanking the HO-site. MM3MG cells and cells overexpressing TLK1B-KD (kinase-dead) were compared for kinetics of DSB repair. For greater sensitivity, the PCR reaction included [α-^32^P]dATP, and 23 cycles were employed. FABP1 (fat binding protein 1) is a control product from a single copy gene. C) *Western blot of E3::HO*. This is shown during a time course of Adeno-HO infection.

We have recently described a system in MM3MG cells that allows to study the repair of a single genomic DSB using the yeast HO-nuclease expressed from adenovirus [11]; a diagram of the HO target cassette shown in Fig. 4A. We have repeated here a time course of HO cleavage to reintroduce the system. During a time course of infection, the HO endonuclease generated a single DSB at the HO-site, which could be monitored as a loss of a PCR product from genomic DNA with primers flanking the HO site (amplicon T7/Puro2). At an MOI of 300, the cleavage is complete at 3 h post-infection (PI), after which, repair occurs mostly by high-fidelity religation (Fig. 4B). To control for input DNA, a different amplicon was generated from the single-copy FABP1 gene on chromosome 6. This time-course of repair coincides with the pattern of expression of HO endonuclease, which peaks after 1 h and is then largely degraded by 3 h PI (Fig. 4C), in both control or KD-expressing cells. We have also reported that in the isogenic MM3MG cells overexpressing TLK1B or the KD protein, the progress of DSB repair was somewhat faster [11]. This was reproduced here (compare the 6 h time-point), and it confirmed that the kinase activity of TLK1B is not required for all the functions of this protein in DSB repair. Of course, the kinase is absolutely required for the phosphorylation of Rad9 at S328 and that of Asf1. We should also mention that, despite the fact that the KD-expressing cells can repair the HO-mediated cleavage with comparable or even faster kinetics than the control cells, they are nonetheless very sensitive to IR-mediated DSBs [4]. This may be related to the incapacity to phosphorylate Rad9 [11]. We have reported that cells overexpressing TLK1B or the KD recruited Rad9 in proximity of the DSB to much greater level than control cells, consistent with the specific interaction of TLK1B with Rad9. We postulated that the TLK1B-Rad9 interaction promotes and/or stabilizes their association with the DSB. Since it is the Rad17-RFC clamp-loader that locks the 9-1-1 complex onto damaged DNA [25], we wanted to test if the occupancy of Rad17 in proximity of the DSB was altered in KD-overexpressing cells. We reasoned that the recruitment of Rad17 to the DSB should not be affected by the KD, as there is no direct interaction of Rad17 with TLK1B, and further the binding of the clamp loader to DSBs precedes 9-1-1 assembly. On the other hand, it seemed possible that the phosphorylation of Rad9 cells could somehow affect the association or stability of the Rad17-RFC/9-1-1 complex with the DSB.

#### The dissociation of Rad17 and Rad9 is delayed after repair in KD-expressing cells and may affect viability

**Figure 5:**
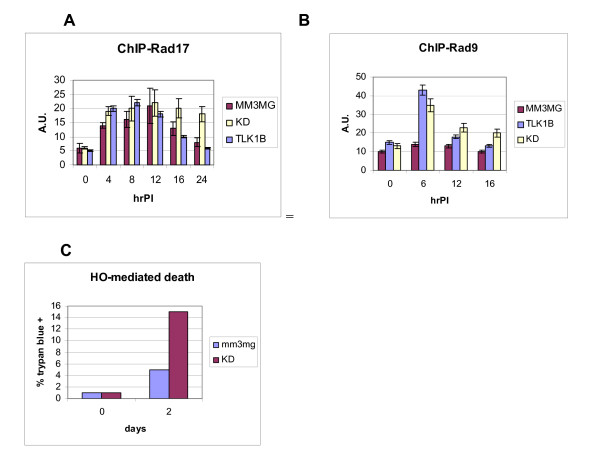
**Association of Rad17 clamp-loader and Rad9 at chromatin adjacent a DSB**. Conditions for ChIP were described in [11]. The occupancy of Rad17 (A) and Rad9 (B) adjacent to the DSB was monitored during a time course of adeno-HO infection, using primers puro1/puro2. The signals of the bands (quantitated with Image J) were normalized to that of the uncleaved DNA at the FABP1 locus. The average of the data and error-bars from 3 complete experiments are shown. C) *HO-mediated death in control and TLK1B-KD-expressing cells*. Cells were infected with adeno-HO, and either immediately collected or collected two days later. The cells were stained with Trypan blue and counted to monitor viability.

We tested this by ChIP for Rad17 and primers Amp1/Amp2 on the immediate left side of the DSB (Fig. 5A). The association of Rad17 was somewhat faster in TLK1B and KD-overexpressing cells, perhaps comparable to the faster repair kinetics (compare with Fig. 4B), but this was not considered very significant. However, there was a significant retardation in the loss of occupancy of Rad17 at 16 and 24 h PI in the KD cells. This was of interest because at that time point, the repair of the DSB appears complete. However, it showed that Rad17 remains associated at the repaired junctions. We also found by ChIP that the release of Rad9 associated near the DSB was also retarded in KD-expressing cells compared to the control (Fig. 5B). In contrast, cells expressing wt-TLK1B showed near complete loss of Rad9 occupancy by 12 h PI. A possible explanation for this result is that perhaps the phosphorylation of Rad9 after DSB repair has a function in release of the clamp-loader/9-1-1 complex and in recovery from damage. This could impact also the re-entry into the cell cycle. Cells expressing the KD show a loss of Rad9 phosphorylation as indicated by a change in mobility, with and without IR [11]. Furthermore, ES-Rad9-/- cells complemented with mut-Rad9(S328A) show defects in cell cycle recovery from IR, in contrast to cells complemented with wt-Rad9 [11]. We thus, decided to test the viability of KD-expressing cells two days after infection with Adeno-HO, a time when we know that the majority of the cells have repaired the DSB. Interestingly, ~15% of the KD cells stained with Trypan blue (dead) at 2 days PI, in contrast to only ~5% of control cells (Fig. 5C). This indicates that, even though the DSB is repaired, some cells do not recover and die. Note that the KD cells are perfectly viable and do not show dead cells in the absence of DNA damage, even though they are partly aneuploid [4]. We should also point out that the repair kinetics of IR-induced DSBs is generally faster than the HO-induced cleavage due to persistence of the enzyme for 3 h, so that it should not be surprising that a single genomic DSB can result in loss of viability due apoptotic activation. It is tempting to speculate that this greater loss of viability in KD cells is due to a late effect of Rad9 phosphorylation on cell cycle recovery after DSB induction. Rad9 is involved in signaling to ATM/ATR and Chk1 and in mediating cell cycle arrest [45] that can be important to allow time for repair. However, re-entry into the cell cycle is also essential for recovery and avoiding apoptosis; hence a possible involvement of the clamp-loader/9-1-1. Of course, we cannot rule out the alternative explanation that 15% of the KD-expressing cells fail to repair the DSB and thus die. However, such interpretation is more difficult to reconcile mechanistically with the more rapid association of Rad17 and Rad9 at the DSB in these cells, if this is indeed the reason for the more rapid repair. To restate this, the major effect seen in KD-expressing cells was rather a delay in dissociation of Rad17 and Rad9 at the repaired junctions - not a delay in repair of the DSB. This is actually in line with recent work that suggests that Rad9 and Rad17 persist at IR-generated foci beyond the time required for the actual repair, and until the genotoxic stress has ceased and the cell cycle resumes, a process mediated by ATR-dependent phosphorylation of Rad17 [46].

#### The phosphorylation of Rad9-S328 fluctuates during repair of DSBs

**Figure 6:**
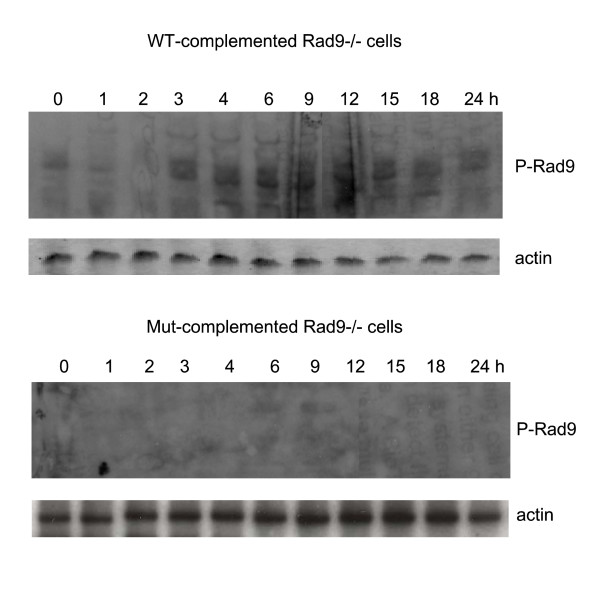
**The phosphorylation of Rad9-S328 fluctuates during repair of DSBs**. ES-Rad9-/- cells complemented with WT and S328A-Rad9 were irradiated (5 Gy), and immediately processed (t = 0) or allowed to recovery for the indicated hours. The blots were sequentially probed with an antiserum for P-Rad9 and for actin.

An antibody for phospho-S328-Rad9 has recently become available, allowing us to test the pattern of P-Rad9 after the generation of DSBs. We reasoned that a single DSB generated with adeno-HO may be insufficient to alter the overall state of P-Rad9. Hence, we used our Rad9-/- cells complemented with WT and S328A-Rad9 [11] to test for possible alterations in P-Rad9 during a time course of recovery from IR. The P-Rad9 signal dropped during the first 2 h after IR, and then increased at later time points, reaching its maximum at 6 h and exceeding the signal obtained for the t = 0. The signal was progressively diminished starting at 15 h after IR, and returning to control levels by 18 h (Fig. 6). As expected, the cells complemented with the Rad9(S328A) mutant did not show a signal for P-Rad9.

## The Working Model

**Figure 7:**
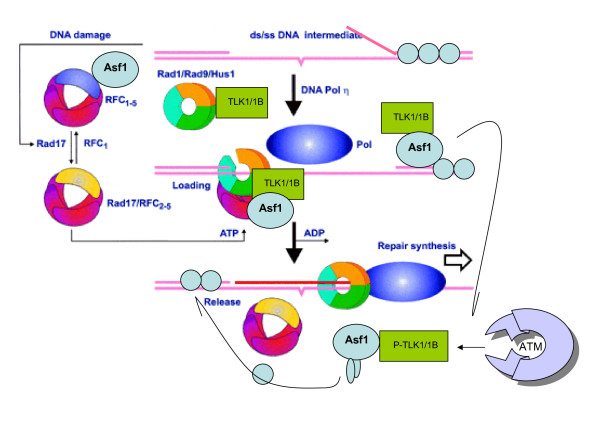
**Model for the activity of TLK1/1B in translesion repair**. Rad9 is known to be involved in translesion repair synthesis [34]. TLK1B helps modulating the activity and assembly of the 9-1-1 complex and promoting repair-coupled chromatin remodeling which depends on Asf1. Integration of the two activities is that TLK1/1B is first recruited to a DSB in a complex with 9-1-1 and the RFC-Rad17 clamp loader, to which Asf1 also binds [47]. At this point, TLK1/1B exchanges with Asf1 to promote nucleosomes eviction and access of the repair machinery to unencumbered DNA [11]. Faster repair can thus take place and is also followed by more rapid reassembly of chromatin, which is believed to be the real signal for resumption of the cell cycle [39]. We suggest that in DSB repair, Rad9 activity on ends-processing has an even more important role in repair [51].

Asf1 is known to interact with RFC (subunits 2-5) tethered to PCNA, and it is recruited to the replication forks [47] We propose that after DNA damage, Asf1 is similarly recruited to the lesions to prepare for repair. In this capacity, Asf1 may be instead recruited by the Rad17-RFC clamp-loader, just as Rad9 is, in association with TLK1/1B. After dissociation from RFC [47], the recruited Asf1 is positioned to disrupt the H3/H4 tetramer resulting in nucleosome eviction. As repair progresses, newly synthesized TLK1B induced by DNA damage, or TLK1B already overexpressed, leads to dissociation of the Asf1/H3/H4 heterotrimer thus promoting the formation of the core tetramer [44]. The H3/H4 tetramer may then be redeposited onto repaired DNA by the HIR complex [48] without necessarily Asf1 participation (Fig. 7). Many details remain to be filled-in - for instance the role that ATM plays in modulating the two separate activities of TLK1 (kinase and chaperone). We recently showed that the association of TLK1B with Asf1 is regulated by its phosphorylation [44]. Thus, a possible outcome for the role of ATM-mediated inhibition of TLK1/1B is that the resulting reduction of Asf1 phosphorylation would lead to a more stable association of TLK1/1B-Asf1, instead of a kinetic association between the two proteins involving the ratio of unphosphorylated and phosphorylated Asf1. This could also lead to dissociation of the Asf1/H3/H4 heterotrimer. Another question is how the Rad9-mediated checkpoint activation of ATM and ATR may affect the entire pathway and its own association with TLK1/1B and Rad17. In addition, since TLK1 kinase activity is rapidly inhibited after DSBs, this could result in accumulation of dephosphorylated (S328) Rad9. After TLK1/1B kinase activity is restored after repair, Rad9 may then be re-phosphorylated, which could be an important mark for release of the clamp complex and signaling completion of repair and resumption of the cell cycle.

## Conclusions

In the past few years, significant evidence has emerged indicating that TLKs are involved in DNA repair [3,15,17]. The mechanism of repair was held to be mainly indirect, and modulated by the known interaction of TLK1 with Asf1 [9]. However, more recently Rad9 was identified as a critical interacting partner of TLK1B [11]. This has opened the door for different studies of the function of TLK1/1B as a direct mediator of DSB repair via processing of the ends, which is a critical function attributed to Rad9 (or 9-1-1). Indeed, Rad9 was critical in stimulating processing and repair of cut plasmids with incompatible ends; and we previously demonstrated that Rad9 is essential for the stimulation of DSB repair by TLK1B, since overexpression of TLK1B alone in Rad9-/- ES cells does not restore repair [11]. We should stress, however, that in our in vitro repair conditions, we start the reactions with naked DNA, and that the formation of chromatin on the plasmid is detected only after the plasmid is reclosed and becomes supercoiled. This situation is quite different than that in intact cells, where the repair machinery must contend with chromatin [49]. In such conditions, the role played by Asf1 may be quite different, and likely more important, in disassembly of nucleosomes to promote access of the repair machinery to unencumbered DNA [11,49]. TLK1B displays two activities on Rad9: phosphorylation, which may affect the checkpoint role of Rad9, and chaperone activity, which may assist its recruitment to DSBs and resulting in ends processing. Although we were not able previously to assign a specific role for the phosphorylation of Rad9 by TLK1B, we have now shown that expression of the KD dominant mutant, which reduces the phosphorylation of Rad9 in vivo [11], resulted in a delay in the release of Rad9 and Rad17 from the DSB. Although we were not able to test a model for a change in P-Rad9 with the adeno-HO-infected cells, we were able to confirm a pattern of fluctuation during IR and recovery. Hence, the phosphorylation of S328-Rad9 decreases during the initial period after IR and then recovers during repair. This is consistent with the model proposed above. Overall, this pattern of P-Rad9 fluctuation may be important for restoring cell cycle re-entry and release from the checkpoint induced by the DSB, and improving viability. In fact, KD-expressing cells are very radiosensitive [4].

## Methods

### Preparation of nuclear extract

MM3MG cells (5 × 10^6^) were centrifuged in a swing-out rotor at 150 × *g *for 10 min at 4°C. The pellet was resuspended in 1 ml ice-cold nuclei isolation buffer [50 mM Tris-HCl (pH 7.5), 0.05 mM spermine, 0.125 mM spermidine, 0.5 mM EDTA, 20 mM KCl, 0.1 mM PMSF, 0.1% (v/v) aprotinin, and 1 mM DTT]. After swelling on ice for 10 min, the cells were broken with a dounce homogenizer using a Wheaton B pestle. Nuclei were pelleted by centrifugation at 150 × *g *for 10 min at 4°C. Nuclei were lysed by suspending in ice-cold nuclei extraction buffer [10 mM HEPES (pH 7.5), 350 mM KCl, 0.2 mM EDTA, 3 mM MgCl_2_, 0.1 mM DTT, 0.2 mM PMSF, 0.2% aprotinin (v/v), and 15% (v/v) glycerol]; and incubated on ice for 30 min. The nuclear envelopes were then removed at 70,000 × *g *for 20 min at 4°C.

### Antibodies, ChIP, and reagents

Rad9, Asf1, and Ku70 antibodies were purchased from Santa Cruz Biotechnology. TLK1/1B antiserum was made in our lab. Anti-E3-11.6k (fusion protein with HO) was a kind gift of Drs. Tollefson and Wold (St. Louis University). P-S328-Rad9 antiserum was from Abgent. Asf1 siRNAs were from Dharmacon. Recombinant Rad9 and TLK1B were prepared as previously described [15]. The MM3MG cells overexpressing TLK1B or the KD were previously described [15], as well as conditions for adeno-HO infection and ChIP adjacent the DSB [11].

The supernatant was retained and dialyzed for 1 h against E buffer [20 mM Tris-HCl (pH 8.0), 0.1 mM KOAc, 20% (v/v) glycerol, 0.5 mM EDTA, and 1 mM DTT], fast frozen, and stored at -80°C.

### In vitro repair

Typical reactions contained 1-2 μg of plasmid; 5-10 μg of nuclear extract; and an energy mix [15,3].

## Authors' contributions

CC and ADB contributed Figure 1 and Figure 2. ADB contributed Figure 3 and 4. JR contributed Figure 5. ADB contributed Fig. 6 and the model (Fig. 7). ADB wrote the paper with the help of CC and JR. All authors have read and approved the final version of the manuscript.
